# Supplementary material for: Non-invasive imaging reveals conditions that impact distribution and persistence of cells after in vivo administration
Source: Stem Cell Res Ther. 2018 Nov 28;9:332. doi: 10.1186/s13287-018-1076-x (PMC6264053; doi:10.1186/s13287-018-1076-x)
Supplement: Supplementary file 1 — Summary of registered clinical studies involving umbilical cord-derived MSCs. (PDF 374 kb) [file 13287_2018_1076_MOESM1_ESM.pdf]

Additional File 1. Summary of registered clinical studies involving umbilical cord-derived MSCs.

Title	Start Date	URL
Pilot Study of Umbilical Cord Blood Transplantation in Adult Patient With Advanced Hematopoietic Malignancies	Oct-02	NCT00514722
Safety and Efficacy Study of Umbilical Cord Blood-Drived Mesenchymal Stem Cells to Promote Engraftment of Unrelated Hematopoietic Stem Cell Transplantation	Aug-08	NCT00823316
Allogeneic Umbilical Cord Mesenchymal Stem Cell Transplantation for Type 1 Diabetes With Diabetic Ketoacidosis	Jan-09	NCT02763423
Safety and Efficacy of Stem Cell Therapy in Patients With Autism	Mar-09	NCT01343511
Safety and Efficacy of Human Mesenchymal Stem Cells for Treatment of Liver Failure	Mar-09	NCT01218464
Umbilical Cord Mesenchymal Stem Cells for Patients With Liver Cirrhosis	May-09	NCT01220492
Allogeneic Mesenchymal Stem Cell for Graft-Versus-Host Disease Treatment	Sep-09	NCT00749164
Mesenchymal Stem Cells Treat Liver Cirrhosis	Oct-09	NCT01233102
Safety and Efficacy of Umbilical Cord Mesenchymal Stem Cell Therapy for Patients With Hereditary Ataxia	Jan-10	NCT01360164
Safety and Efficacy of Umbilical Cord Mesenchymal Stem Cell Therapy for Patients With Progressive Multiple Sclerosis and Neuromyelitis Optica	Jan-10	NCT01364246
Safety and Efficacy Study of Umbilical Cord/Placenta-Derived Mesenchymal Stem Cells to Treat Myelodysplastic Syndromes	May-10	NCT01129739
Intratracheal Umbilical Cord-derived Mesenchymal Stem Cells for Severe Bronchopulmonary Dysplasia	Jul-10	NCT01207869
Safety and Efficacy Study of Umbilical Cord/Placenta-Derived Mesenchymal Stem Cells to Treat Severe Aplastic Anemia	Aug-10	NCT01182662
Stem Cell Therapy for Type 1 Diabetes Mellitus	Aug-10	NCT01143168
Umbilical Cord Mesenchymal Stem Cells Infusion for Ulcerative Colitis	Sep-10	NCT01221428
Umbilical Cord Mesenchymal Stem Cells Infusion for Initial Type 1 Diabetes Mellitus	Sep-10	NCT01219465
Umbilical Cord Mesenchymal Stem Cells Infusion Via Hepatic Artery in Cirrhosis Patients	Oct-10	NCT01224327
Intramuscular Injection of Mesenchymal Stem Cell for Treatment of Children With Idiopathic Dilated Cardiomyopathy	Oct-10	NCT01219452
Human Umbilical Cord Mesenchymal Stem Cells Transplantation for Patients With Decompensated Liver Cirrhosis	Oct-10	NCT01342250
Safety and Efficacy Study of Umbilical Cord/Placenta-Derived Mesenchymal Stem Cells to Treat Ankylosing Spondylitis (AS)	Jan-11	NCT01420432

Umbilical Cord Mesenchymal Stem Cells Injection for Diabetic Foot	Jan-11	NCT01216865
Allogenic Stem Cell Therapy in Patients With Acute Burn	Jul-11	NCT01443689
Safety and Efficacy Study of Umbilical Cord/Placenta-Derived Mesenchymal Stem Cells to Treat Type 2 Diabetes	Jul-11	NCT01413035
Safety and Efficacy of Umbilical Cord Mesenchymal Stem Cell Therapy for Patients With Duchenne Muscular Dystrophy	Oct-11	NCT01610440
Umbilical Cord Mesenchymal Stem Cells for Patients With Autoimmune Hepatitis	Oct-11	NCT01661842
Umbilical Cord Mesenchymal Stem Cells for Patients With Primary Biliary Cirrhosis	Oct-11	NCT01662973
A New Method to Treat Hereditary Cerebellar Ataxia - Umbilical Cord Mesenchymal Stem Cells Transplantation	Dec-11	NCT01489267
Umbilical Cord Derived Mesenchymal Stem Cells Transplantation for Active and Refractory Systemic Lupus Erythematosus	Jan-12	NCT01741857
Umbilical Cord Blood-derived Mesenchymal Stem Cells for the Treatment of Steroid-refractory Acute or Chronic Graft-versus-host-disease	Jan-12	NCT01549665
UC-MSCs Gel Treatment Difficult Healing of Skin Ulcers	Jan-12	NCT02685722
MSCs Source of Sweat Gland Cells of Large Area Skin Injury Patients Transplant of the Wound	Jan-12	NCT02669199
Phase 2 Study of Human Umbilical Cord Derived Mesenchymal Stem Cell for the Treatment of Lupus Nephritis	Feb-12	NCT01539902
Human Mesenchymal Stem Cells Induce Liver Transplant Tolerance	Feb-12	NCT01690247
Safety and Efficiency of Umbilical Cord-derived Mesenchymal Stem Cells(UC-MSC) in Patients With Alzheimer's Disease	Mar-12	NCT01547689
The Clinical Trial on the Use of Umbilical Cord Mesenchymal Stem Cells in Amyotrophic Lateral Sclerosis	Mar-12	NCT01494480
Stem Cell Therapy Combined Hormone Replacement Therapy in Patients With Premature Ovarian Failure	Mar-12	NCT01742533
Umbilical Cord Mesenchymal Stem Cell Treatment for Crohn's Disease	Jun-12	NCT02445547
Safety and Efficacy of Human Umbilical Cord Derived Mesenchymal Stem Cells for Treatment of HBV-related Liver Cirrhosis	Sep-12	NCT01728727
Umbilical Cord Mesenchymal Stem Cells Transplantation Combined With Plasma Exchange for Patients With Liver Failure	Nov-12	NCT01724398
Safety and Efficacy of UC-MSC in Patients With Acute Severe Graft-versus-host Disease	Dec-12	NCT01754454
Randomized Clinical Trial of Intravenous Infusion Umbilical Cord Mesenchymal Stem Cells on Cardiopathy	Dec-12	NCT01739777
Mesenchymal Stem Cells to Treat Type II Diabetes	Jan-13	NCT02302599

Umbilical Cord Mesenchymal Stem Cells for Immune Reconstitution in HIV-infected Patients	Jan-13	NCT01213186
Safety and Efficacy Study of Umbilical Cord-Derived Mesenchymal Stem Cells for Rheumatoid Arthritis	Apr-13	NCT01547091
Safety and Efficacy of Diverse Mesenchymal Stem Cells Transplantation for Liver Failure	Jul-13	NCT01844063
Umbilical Cord Derived Mesenchymal Stromal Cells For The Treatment of Severe Steroid-resistant Graft Versus Host Disease	Sep-13	NCT02032446
Umbilical Cord Derived Mesenchymal Stem Cells Therapy in Hypoxic Ischemic Encephalopathy	Sep-13	NCT01962233
Umbilical Cord Tissue-derived Mesenchymal Stem Cells for Rheumatoid Arthritis	Oct-13	NCT01985464
Umbilical Cord Derived Mesenchymal Stem Cells Therapy in Ischemic Cardiomyopathy	Oct-13	NCT01946048
Treatment Protocol of Child SAA With the Injection of Umbilical Cord Derived Mesenchymal Stem Cells	Oct-13	NCT02218437
Efficacy of Umbilical Cord Mesenchymal Stem Cells in Duchenne Muscular Dystrophy	Nov-13	NCT02285673
Safety and Feasibility Study of Mesenchymal Trophic Factor (MTF) for Treatment of Osteoarthritis	Dec-13	NCT02003131
Umbilical Cord Mesenchymal Stem Cells Transplantation to Patients With Spinal Cord Injury	Jan-14	NCT02481440
Feasibility Study of Human Umbilical Cord Tissue-Derived Mesenchymal Stem Cells in Patients With Multiple Sclerosis	Jan-14	NCT02034188
Umbilical Cord Mesenchymal Stem Cells Injection for Diabetes Secondary Peripheral Arterial Disease	Feb-14	NCT02287831
Allogeneic Umbilical Cord Mesenchymal Stem Cell Therapy for Autism	Jul-14	NCT02192749
Safety and Feasibility Study of Intranasal Mesenchymal Trophic Factor (MTF) for Treatment of Asthma	Jul-14	NCT02192736
Mesenchymal Stem Cells Transplantation for Ischemic-type Biliary Lesions	Jul-14	NCT02223897
Allogenic Mesenchymal Stem Cell for Bone Defect or Non Union Fracture	Aug-14	NCT02307435
Allogeneic Human Umbilical Cord Mesenchymal Stem Cells for a Single Male Patient With Duchenne Muscular Dystrophy (DMD)	Sep-14	NCT02235844
Clinical Study of Umbilical Cord Tissue Mesenchymal Stem Cells (UC-MSC) for Treatment of Osteoarthritis	Sep-14	NCT02237846
Safety and Feasibility Study of Cell Therapy in Treatment of Spinal Cord Injury	Sep-14	NCT02237547
A Study on Radiation-induced Pulmonary Fibrosis Treated With Clinical Grade Umbilical Cord Mesenchymal Stem Cells	Oct-14	NCT02277145
Treatment of Infertility by Collagen Scaffold Loaded With Umbilical Cord Derived Mesenchyma Stem Cells	Nov-14	NCT02313415

Human Umbilical Cord Mesenchymal Stem Cell Transplantation in Articular Cartilage Defect	Dec-14	NCT02291926
Human Umbilical Cord Stroma MSC in Myocardial Infarction	Feb-15	NCT02323477
A Study of Allogeneic Human UC-MSC and Liberation Therapy (When Associated With CCSVI) in Patients With RRMS	Feb-15	NCT02587715
A Study of Allogeneic Human UC-MSC and Liberation Therapy (When Associated With CCSVI) in Patients With RRMS	Feb-15	NCT02418325
Evaluation of the Safety and Potential Therapeutic Effects After Intravenous Transplantation of Cordstem-ST in Patients With Cerebral Infarction	Feb-15	NCT02378974
Human Umbilical Cord Mesenchymal Stem Cell in Cerebral Hemorrhage Sequela	Mar-15	NCT02283879
Safety and Efficacy of UC-MSCs in Patients With Psoriasis Vulgaris	Apr-15	NCT02491658
Human Umbilical-Cord-Derived Mesenchymal Stem Cell Therapy in Acute Lung Injury	May-15	NCT02444455
Human Umbilical-Cord-Derived Mesenchymal Stem Cell Therapy in Ischemic Cardiomyopathy	May-15	NCT02439541
Human Umbilical-Cord-Derived Mesenchymal Stem Cell Therapy in Active Ulcerative Colitis	May-15	NCT02442037
Human Umbilical-Cord-Derived Mesenchymal Stem Cell Therapy in Paraquat Poisoning Induced Lung Injury	May-15	NCT02444858
Efficacy of Stem Cell Therapy in Ambulatory and Non-ambulatory Children With Duchenne Muscular Dystrophy - Phase 1-2	Jun-15	NCT02484560
Collagen Scaffolds Loaded With HUCMSCs for the Improvement of Erectile Function in Men With Diabetes	Sep-15	NCT02579148
Injectable Collagen Scaffoldâ„¢ Combined With HUC-MSCs for the Improvement of Erectile Function in Men With Diabetes	Sep-15	NCT02745808
Human Umbilical Cord-derived Mesenchymal Stem Cells With Injectable Collagen Scaffold Transplantation for Chronic Ischemic Cardiomyopathy	Oct-15	NCT02635464
Transplantation of HUC-MSCs With Injectable Collagen Scaffold for POF	Oct-15	NCT02644447
A Study to Assess Safety and Efficacy of Umbilical Cord-derived Mesenchymal Stromal Cells in Knee Osteoarthritis	Dec-15	NCT02580695
Umbilical Cord Mesenchymal Stem Cells Infusion for Diabetes Related Vascular Complications	Jan-16	NCT02834858
Human Umbilical Cord-Mesenchymal Stem Cells for Rheumatoid Arthritis	Jan-16	NCT02643823
A Study on Pneumoconiosis Treated With Whole-lung Lavage Combined With Mesenchymal Stem Cells	Jan-16	NCT02668068
Stem Cell Therapy Combined With NeuroRegen Scaffold in Patients With Erectile Dysfunction After Rectal Cancer Surgery	Jan-16	NCT02648386
Umbilical Cord Derived Mesenchymal Stem Cells Treatment in Ischemic Stroke	Feb-16	NCT02580019

Human Umbilical Cord Mesenchymal Stem Cells (HUC-MSCs) Transplantation in Women With Primary Ovarian Insufficiency (POI)	Feb-16	NCT03033277
Human Umbilical Cord-Mesenchymal Stem Cells for Hepatic Cirrhosis	Mar-16	NCT02652351
Stem Cells for Treatment of Bronchopleural Fistula	Apr-16	NCT02961725
Safety and Exploratory Efficacy Study of UCMSCs in Patients With Ischemic Heart Disease (SEESUPIHD)	May-16	NCT02666391
Injectable Collagen Scaffold Combined With HUC-MSCs Transplantation for Patients With Decompensated Cirrhosis	May-16	NCT02786017
Safety and Exploratory Efficacy Study of Collagen Membrane With Mesenchymal Stem Cells in the Treatment of Skin Defects	May-16	NCT02672280
Human Umbilical Cord-Mesenchymal Stem Cells for Pneumoconiosis	Jun-16	NCT02790762
UC-MSC Infusion for HBV-Related Acute-on-Chronic Liver Failure	Jun-16	NCT02812121
UCMSC Transplantation in the Treatment of Cartilage Damage	Jun-16	NCT02776943
MsciSLE: MSCs in SLE Trial	Jul-16	NCT02633163
Safety and Efficacy of Umbilical Cord Mesenchymal Stem Cell Local Intramuscular Injection for Treatment of Uterine Scars	Nov-16	NCT02968459
Collagen Membrane Combined With HUC-MSCs Transplantation in Patients With Nasal Septum Perforation	Nov-16	NCT02947191
Safety Study of Filler Agent Composed of Umbilical Cord Mesenchymal Stem Cells and Hyaluronic Acid	Dec-16	NCT02698813
Umbilical Cord Derived Mesenchymal Stem Cells Therapy in Aplastic Anemia	Jan-17	NCT03055078
Umbilical Cord Mesenchymal Stem Cells in Primary Sclerosing Cholangitis	Jan-17	NCT03516006
Phase I Mesenchymal Stem Cells for Systemic Lupus Erythematosus	Apr-17	NCT03171194
hCT-MSCs for Children With Autism Spectrum Disorder (ASD)	Jun-17	NCT03099239
A Single-arm,Phase IIa,Safety and Efficacy Trial of Selected MSCs in the Treatment of Patients With PSC & AiH	Jun-17	NCT02997878
Human Umbilical Cord Mesenchymal Stem Cell Therapy for Cerebral Infarction Patients in Convalescent Period.	Jul-17	NCT03176498
The Study of Heart Failure With Human Umbilical Cord Mesenchymal Stem Cells (hUC-MSC)	Jul-17	NCT03180450
The Study of Early Stage Osteonecrosis of Femoral Head With Human Umbilical Cord Mesenchymal Stem Cells	Jul-17	NCT03180463
The Safety and Efficacy of Human Umbilical Cord Mesenchymal Stem Cells in the Treatment of Acute Cerebral Infarction	Jul-17	NCT03186456
Research for Human Umbilical Cord Mesenchymal Stem Cells in the Treatment of Myelodysplastic Syndrome (MDS)	Jul-17	NCT03184935

Umbilical Cord Derived Mesenchymal Stem Cells Therapy in Systemic Lupus Erythematosus	Aug-17	NCT03219801
Allogenic Mesenchymal Stem Cells And Physical Therapy for MS Treatment	Sep-17	NCT03326505
Safety and Exploratory Efficacy Study of UCMSCs in Patients With Alzheimer's Disease	Oct-17	NCT02672306
Safety of Umbilical Cord Mesenchymal Stem Cell Local Intramuscular Injection for Treatment of Uterine Scars	Nov-17	NCT03181087
Intrathecal Transplantation of UC-MSC in Patients With Late Stage of Chronic Spinal Cord Injury	Jan-18	NCT03505034
Evaluating Safety and Efficacy of Mesenchymal Stem Cells From Umbilical Cord	Jan-18	NCT03358654
Intrathecal Transplantation of UC-MSC in Patients With Early Stage of Chronic Spinal Cord Injury	Jan-18	NCT03521323
Intrathecal Transplantation of UC-MSC in Patients With Sub-Acute Spinal Cord Injury	Jan-18	NCT03521336
Umbilical Cord Mesenchymal Stem Cells Injection for Ocular Corneal Burn	Jan-18	NCT03237442
The Safety/Efficacy of Umbilical Cord Mesenchymal Stem Cell Therapy for Patients With Healing Poor After Uterus Injury	Jan-18	NCT03386708
The Safety/Efficacy of Human Umbilical Cord Mesenchymal Stem Cells Therapy for Patients With Osteoarthritis	Jan-18	NCT03383081
The Maximum Tolerated Dose of Mesenchymal Stem Cells From Umbilical Cord	Jan-18	NCT03357770
Repair of Acute Respiratory Distress Syndrome by Stromal Cell Administration (REALIST)	Mar-18	NCT03042143
Assessement the Reconstruction of Motor Circuits in Nerve Fiber Injuries After the Treatment of Umbilical Cord Mesenchymal Stem Cells With Blood Oxygen Level-dependent Drived Diffusion Tensor Imaging	Mar-18	NCT03336996
Transplantation of Umbilical Cord-derived Mesenchymal Stem Cells Via Different Routes	Apr-18	NCT03414697
Intravenous Infusion of Umbilical Cord Tissue (UC) Derived Mesenchymal Stem Cells (MSCs) Versus Bone Marrow (BM) Derived MSCs to Evaluate Cytokine Suppression in Patients With Chronic Inflammation Due to Metabolic Syndrome.	Apr-18	NCT03059355
Clinical Trial of Umbilical Cord Mesenchymal Stem Cell Transfusion in Decompensated Liver Cirrhosis	Jun-18	NCT03529136
Clinical Study of Umbilical Cord Mesenchymal Stem Cells (UC-MSC) for Treatment of Knee Osteoarthritis	Oct-18	NCT03166865
