# Supplementary material for: Non-invasive imaging reveals conditions that impact distribution and persistence of cells after in vivo administration
Source: Stem Cell Res Ther. 2018 Nov 28;9:332. doi: 10.1186/s13287-018-1076-x (PMC6264053; doi:10.1186/s13287-018-1076-x)
Supplement: Supplementary file 2 — Culture media used for growing cells. (PDF 326 kb) [file 13287_2018_1076_MOESM2_ESM.pdf]

**Additional File 2.** Culture media used for growing cells.

| | |
|---|---|
| mKSCs, mMSCs, RAW macrophages, hBM-MSCs: | Dulbecco's Modified Eagle's Medium (DMEM, D6546, Sigma), 10% Foetal Calf Serum (FCS), 2 mM L-Glutamine. |
| hKCs: | DMEM/F12 (12-719Q, Lonza), 5% FCS, epidermal growth factor (10 ng/ml), hydrocortisone (36 ng/ml) 3,3',5-Triiodo-l-thyronine sodium salt (4 pg/ml), insulin-transferrin-selenium liquid medium (1x). |
| hUC-MSCs: | Minimum Essential Medium (Life Technologies 32561037), 10% FCS, 5 ng/mL recombinant human basic Fibroblast Growth Factor. |
