# Supplementary material for: Non-invasive imaging reveals conditions that impact distribution and persistence of cells after in vivo administration
Source: Stem Cell Res Ther. 2018 Nov 28;9:332. doi: 10.1186/s13287-018-1076-x (PMC6264053; doi:10.1186/s13287-018-1076-x)
Supplement: Supplementary file 3 — Detailed method of ultrasound-guided intracardiac injection of cells. (PDF 772 kb) [file 13287_2018_1076_MOESM3_ESM.pdf]

Additional File 3. Detailed method of ultrasound guided intracardiac injection of cells.

1. Administer 0.1mg/kg buprenorphine (SC) for pain relief.
2. Anaesthetise mouse with isoflurane and oxygen, and position supine on a heated platform.
3. Remove fur from the chest area using an electric shaver and a depilatory cream (Veet).
4. Tape the limbs into the extended position, and place tape over the abdomen to hold the skin over the chest taut (Fig. 1a).
5. Apply ultrasound gel liberally to the chest, avoiding air bubbles.
6. Position the ultrasound transducer (S-Sharp, Taiwan), over the chest at a slight angle (approx. 30° counter-clockwise) and lower it towards the mouse until the chest is in view (Fig. 1b).
7. Move the platform to a slight downward angle (approx. 45°) (Fig. 1c) and adjust the position of the platform to enable visualisation of the heart.
8. The right ventricle is very difficult to image, due to its small size, so any images of the heart are most likely of the left ventricle. To confirm, move the transducer to the right (anatomical left), away from the heart until it is out of the imaging plane. Then, move the transducer back to bring the heart back into the imaging plane, thus ensuring imaging of the left cardiac ventricle.
9. Draw 100µl of the cell solution up into a 29G ½ inch insulin syringe, ensuring that the cells were adequately re-suspended, and not too cold immediately prior to administration
10. Introduce the tip of the needle in to the imaging plane, and tap the needle on the skin a few times, to ensure that the needle stays within the imaging plane even when moved.
11. Gently insert the needle in to the heart, and administer the cell solution in a slow, smooth, and controlled manner, only when both the tip of the needle and the heart can be visualised at the same time (Fig. 1d – e, 1g). It may be necessary to adjust the angle of the needle slightly to ensure visualisation of both the needle and the heart. If both cannot be visualised even when the needle is adjusted, withdraw the needle,

adjust the position of the mouse, and re-attempt the injection. It is not advised to attempt the injection more than two or three times.

12. Bioluminescence imaging, to confirm success of the injection, can then be performed immediately after the IC injection, under the same anaesthesia session.
13. The mouse should be recovered from anaesthesia in a heat box, and monitored closely for 24h for signs of adverse effects.

Comments on our experience of performing IC injections

Ultrasound guidance allows the direct visualisation of the needle within the mouse heart (Fig. 1g), allowing the researcher to have confidence in the correct placement of the needle before administering the cell solution[1]. This is an advantage over an alternatively described method of performing IC injections[2], where the position of needle insertion is measured using anatomical features, but the needle is inserted into the heart blindly. Moreover, it is far less invasive than the method described by Arguello and colleagues, where the chest is surgically opened in order to visualise the heart for IC injection[3]. A successful injection with no mis-injections in to the right ventricle or the chest cavity requires careful and accurate placement of the needle, and it is essential the both the heart and needle can be seen in the same imaging plane at the same time, before the cell solution is administered. A smooth and controlled administration of the cell solution is preferable to avoid placing additional volume and pressure stress on the heart and surrounding vasculature, but we have found that very long injection times of 30 s or longer are not necessary.

It is possible for adverse effects to occur following IC administration of cells. The least severe side effect is a loss of balance which results in mice leaning to one side (usually the left) and walking in circles for some time, however this is recoverable and generally wears off after 30 minutes to one hour. More severe side effects include:

(i) Hind limb paralysis, where the mouse loses complete function of the hind legs, and, in our experience, function does not return. This negatively affects the welfare of the mouse.
(ii) ‘Spinning’ where the mouse may twist and spin very violently. As the mouse never recovers from this behaviour it should be sacrificed immediately.

(iii) Death.

In our experience, adverse effects are either observed immediately after the mice wake from anaesthesia, or develop over the first hour. While it is difficult to know the exact cause of adverse effects and death, the most likely causes are embolism resulting from clumped cells lodging in vessels, or damage to the heart, resulting in fatal blood loss. Careful cell preparation to avoid cell clumping, and a steady hand during the injection to avoid damaging the heart, appear to improve the outcome. We found that increasing the volume administered to 200µl, and thus decreasing the density of the cell solution, did not have any effect on the outcome with regards to adverse effects. As a result, we decided to continue administering the smaller volume of 100µl.

1. Salamon, J. & Peldschus, K. Ultrasound-guided intracardial injection and in vivo magnetic resonance imaging of single cells in mice as a paradigm for hematogenous metastases. *Methods in molecular biology (Clifton, N.J.)* **1070**, 203-211 (2014).
2. Campbell, J.P., Merkel, A.R., Masood-Campbell, S.K., Elefteriou, F. & Sterling, J.A. Models of bone metastasis. *Journal of visualized experiments : JoVE*, e4260 (2012).
3. Arguello, F., Baggs, R.B. & Frantz, C.N. A murine model of experimental metastasis to bone and bone marrow. *Cancer research* **48**, 6876-6881 (1988).

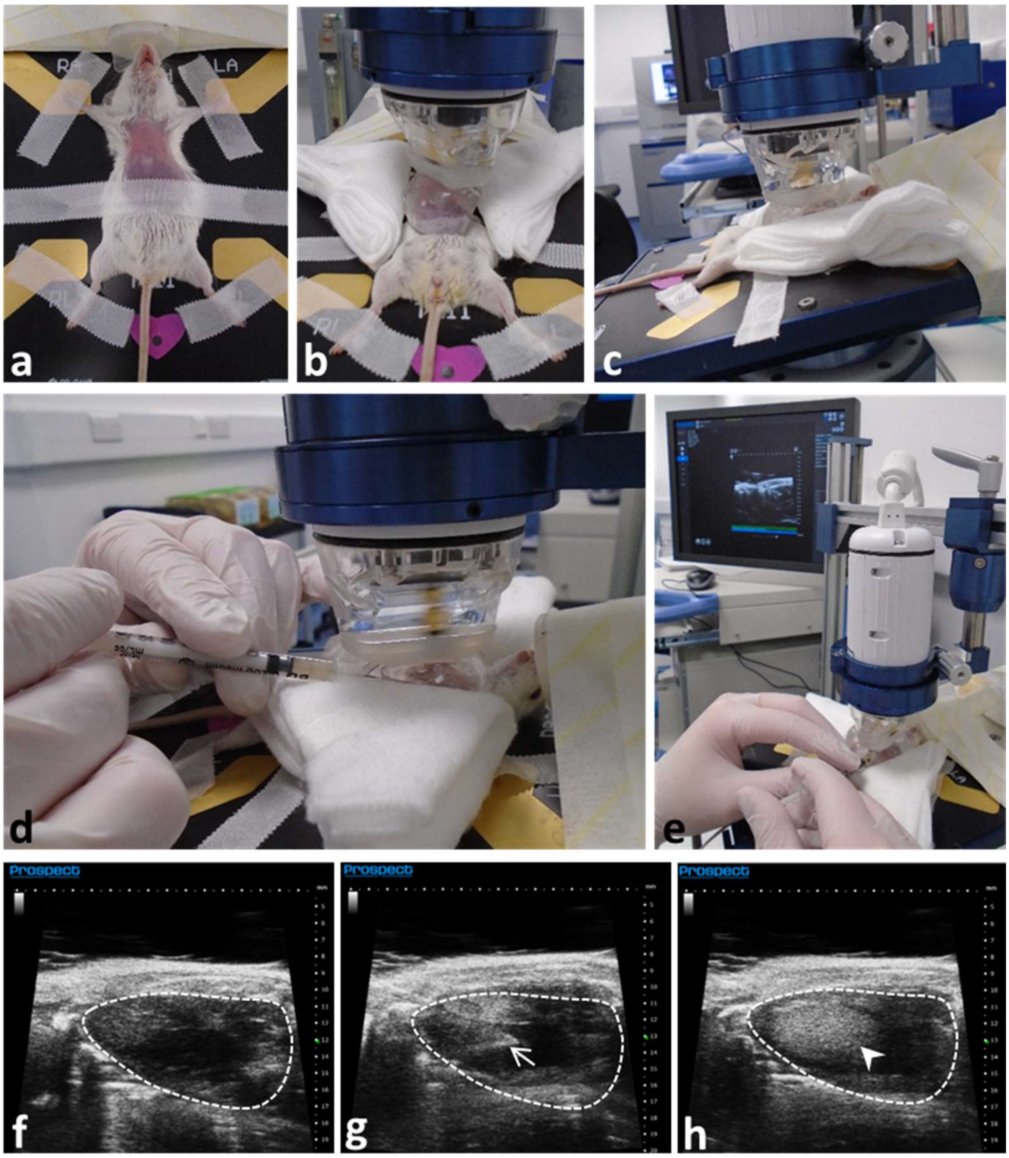

Figure 1. Ultrasound-guided intracardiac administration of cells. (a) Photograph of mouse set-up for ultrasound-guided intracardiac injection. **(b - c)** Photographs of the ultrasound transducer in place for heart imaging. **(d - e)** Photographs of position of needle for intracardiac injection. Snapshot images of left cardiac ventricle, outlined with a dashed white line, **(f)** before injection, **(g)** during the injection, with the tip of the needle (arrow) in the left ventricle and **(h)** after injection of the cell solution, where the contrast within the left ventricle has changed (arrowhead), indicating a successful injection.
