# Supplementary material for: Non-invasive imaging reveals conditions that impact distribution and persistence of cells after in vivo administration
Source: Stem Cell Res Ther. 2018 Nov 28;9:332. doi: 10.1186/s13287-018-1076-x (PMC6264053; doi:10.1186/s13287-018-1076-x)
Supplement: Supplementary file 4 — MSOT images of GNR-labelled macrophages in different organs. Snapshot images of the abdomen of mice, showing the (a) liver, delineated with red lines and (b) kidney, delineated with a yellow line or spleen, delineated with a green line. Images correspond to baseline imaging (pre-administration) and 4 h post IV administration of GNR-labelled RAW macrophages. GNR-labelling generates MSOT contrast, seen as an increase in pixel brightness for areas corresponding to liver and spleen. Kinetic imaging over a period of 4 h was used to generate data shown in Fig. 1f. (c) Quantification of the MSOT mean pixel intensity in the liver, spleen or kidneys 4 h post administration of GNR-labelled RAW macrophages IV or IC. Data is displayed as fold changes in pixel intensity in respect to baseline measurements. (PDF 463 kb) [file 13287_2018_1076_MOESM4_ESM.pdf]

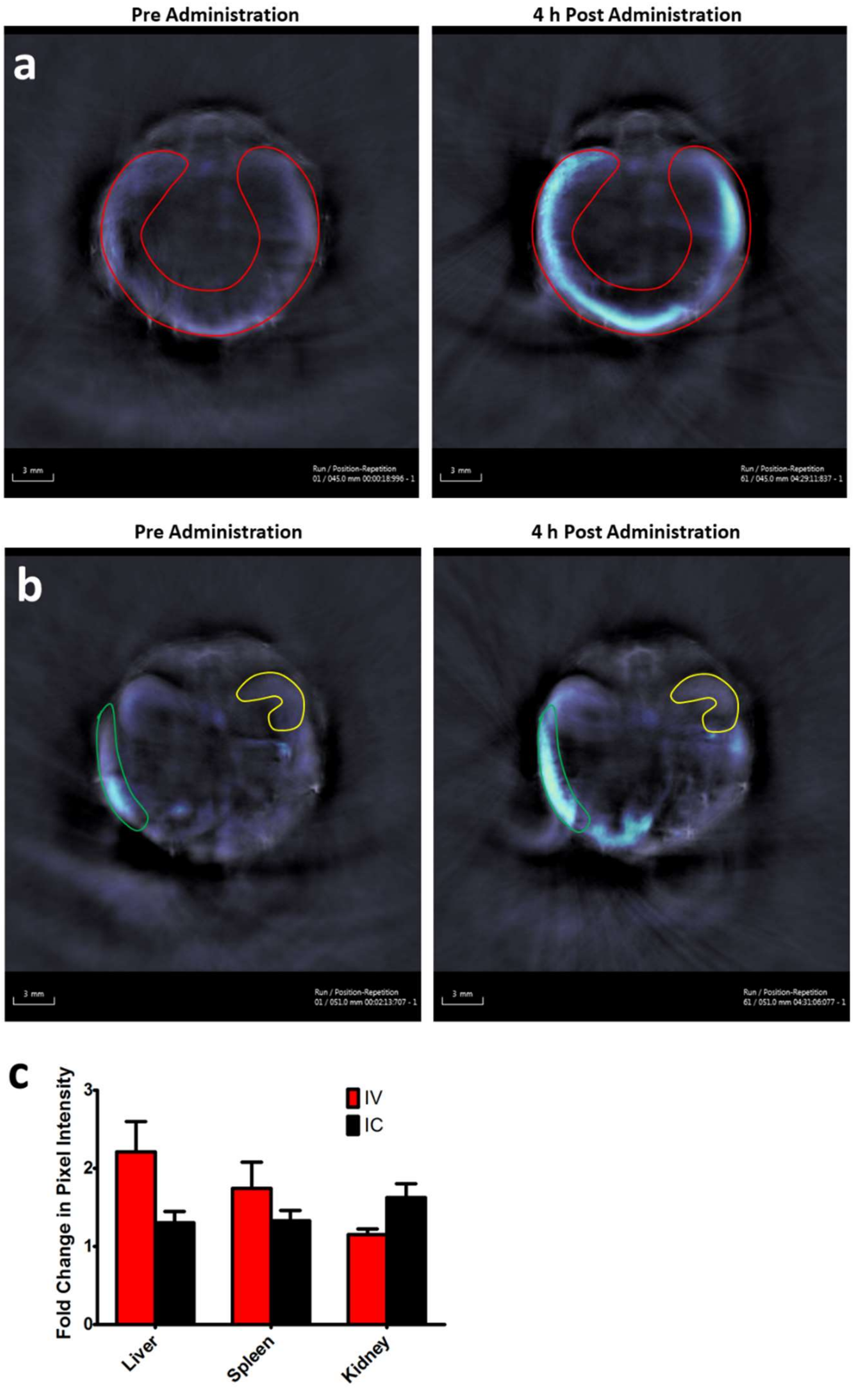

Additional File 4. MSOT images of GNR-labelled macrophages in different organs. Snapshot images of the abdomen of mice, showing the **(a)** liver, delineated with red lines and **(b)** kidney, delineated with a yellow line or spleen, delineated with a green line. Images correspond to baseline imaging (pre-administration) and 4h post IV administration of GNR-labelled RAW macrophages. GNR-labelling generates MSOT contrast, seen as an increase in pixel brightness for areas corresponding to liver and spleen. Kinetic imaging over a period of 4h was used to generate data shown in Fig. 1f. **(c)** Quantification of the MSOT mean pixel intensity in the liver, spleen or kidneys 4h post administration of GNR-labelled RAW macrophages IV or IC. Data is displayed as fold changes in pixel intensity in respect to baseline measurements.
