# Supplementary material for: Non-invasive imaging reveals conditions that impact distribution and persistence of cells after in vivo administration
Source: Stem Cell Res Ther. 2018 Nov 28;9:332. doi: 10.1186/s13287-018-1076-x (PMC6264053; doi:10.1186/s13287-018-1076-x)
Supplement: Supplementary file 5 — Placement of a region of interest (ROI) for the calculation of relaxation time in the kidney’s cortex. (a) In vivo T2*-weighted image of a single kidney post administration of SPION-labelled mMSCs, (b) placement of an ROI (yellow line) covering the cortex of the kidney where cell/SPION contrast is observed and (c) the changes in signal intensity as a function of echo time, with the solid line displaying the exponential fit of the data, from where the relaxation time is derived. Relaxation times were calculated with Paravision 6.0.1. (PDF 462 kb) [file 13287_2018_1076_MOESM5_ESM.pdf]

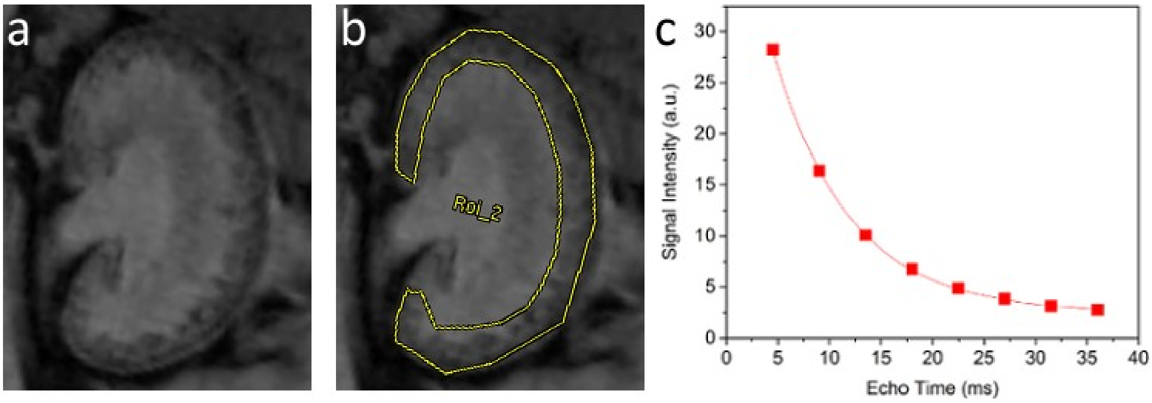

Additional File 5. Placement of a region of interest (ROI) for the calculation of relaxation time in the kidney's cortex. (a) *In vivo* T_2^*-weighted image of a single kidney post administration of SPION-labelled mMSCs, **(b)** placement of an ROI (yellow line) covering the cortex of the kidney where cell/SPION contrast is observed and **(c)** the changes in signal intensity as a function of echo time, with the solid line displaying the exponential fit of the data, from where the relaxation time is derived. Relaxation times were calculated with Paravision 6.0.1.
