# Supplementary material for: Non-invasive imaging reveals conditions that impact distribution and persistence of cells after in vivo administration
Source: Stem Cell Res Ther. 2018 Nov 28;9:332. doi: 10.1186/s13287-018-1076-x (PMC6264053; doi:10.1186/s13287-018-1076-x)
Supplement: Supplementary file 6 — MRI sequences and acquisition parameters. All in vivo data was acquired with a 4-channel surface coil designed for the mouse brain or abdomen. Post mortem data was obtained with a 27 mm volume coil. (PDF 547 kb) [file 13287_2018_1076_MOESM6_ESM.pdf]

Additional File 6. MRI sequences and acquisition parameters. All in vivo data was acquired with a 4-channel surface coil designed for the mouse brain or abdomen. Post mortem data was obtained with a 27 mm volume coil.

	In vivo					***Post Mortem***
	**FLASH T_2* (Abdomen)**	**FLASH T_2* (Brain)**	**B0 Map**	**MGE T_2* Map**	**RARE T_2 (Tumours)**	**FLASH T_2* (Organs)**
**Echo Time**	5.5 ms	4.2 ms	3.6 ms	N/A	25 ms	6.3
**Repetition Time**	262.6 ms	262.6 ms	10 ms	900 ms	2500 ms	1300
**Flip Angle**	20.0°	20.0°	15.0°	50.0°	90° excit. 180° refoc.	20°
**Matrix Size**	386 x 386 pixels	256 x 256 pixels	64 x 64 x 64 pixels	256 x 256 pixels	256 x 192 pixels	386 x 386 pixels
**Field of View**	35 x 35 mm	20 x 20 mm	45 x 45 x 45mm	35 x 35 mm	40 x 30 mm	15 x 15 mm
**Averages**	3	3	3	2	4	24
**Slices**	20	20	1	20	30	70
**Slice Thickness**	0.5 mm	0.5 mm	45 mm	0.5 mm	1.0 mm	0.2 mm
**Echo Images**	N/A	N/A	N/A	8	N/A	N/A
**Echo Spacing**	N/A	N/A	N/A	4.5ms, start at 4.5ms	N/A	N/A
**Acquisition Time**	5m 35s	4m 4s	2m 3s	5m 7s	4m	3h 20m 43s
