# Supplementary material for: Non-invasive imaging reveals conditions that impact distribution and persistence of cells after in vivo administration
Source: Stem Cell Res Ther. 2018 Nov 28;9:332. doi: 10.1186/s13287-018-1076-x (PMC6264053; doi:10.1186/s13287-018-1076-x)
Supplement: Supplementary file 7 — mMSC distribution between day 14 and 30. (a) From day 24 onwards for IC-injected mice, it was necessary to increase the scale by two orders of magnitude (BLI scale 1.0 × 10^7^–1.0 × 10^8^ p/s/cm2/sr, orange frame) compared to that in Fig. 4 to enable visualisation of the very strong signals resulting from rapidly proliferating mMSCs. (b) Using the original scale (see Fig. 4: 1.0 × 10^5^–1.0 × 10^6^ p/s/cm2/sr), signals could be detected by day 24 in one (out of 3) IV-injected mice. (c, e) Representative in vivo and corresponding (d, f) ex vivo organ images at day 30. (d) Small spots of bioluminescence signal could be detected in some of the organs of IC-injected BALB/c SCID mice (arrows), but the scale had to be lowered to 1.0 × 10^4^–1.0 × 10^5^ p/s/cm2/sr (blue frame) in order to be able to display these weak signals. (e) Two out of three IV-administered BALB/c SCID mice did not show any signals at day 30 in vivo using the standard scale (green frame), however, corresponding (f) organ imaging showed small foci of bioluminescence signals in the lungs (arrows). (PDF 618 kb) [file 13287_2018_1076_MOESM7_ESM.pdf]

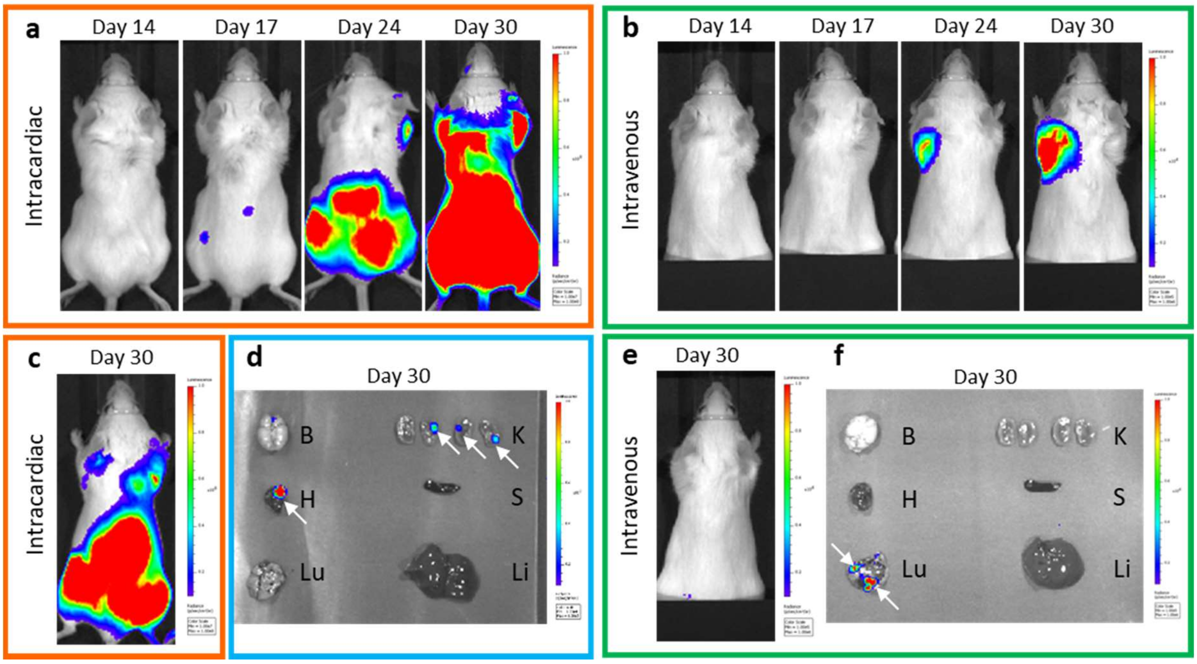

Additional File 7. mMSC distribution between day 14 and 30. (a) From day 24 onwards for IC-injected mice, it was necessary to increase the scale by two orders of magnitude (BLI scale 1.0 x10^7 – 1.0 x10^8 p/s/cm^2/sr, orange frame) compared to that in Fig. 4 to enable visualisation of the very strong signals resulting from rapidly proliferating mMSCs. **(b)** Using the original scale (see Fig. 4: 1.0 x10^5 – 1.0 x10^6 p/s/cm^2/sr), signals could be detected by day 24 in one (out of 3) IV-injected mice. (**c,e**) Representative *in vivo* and corresponding **(d,f)** *ex vivo* organ images at day 30. **(d)** Small spots of bioluminescence signal could be detected in some of the organs of IC-injected BALB/c SCID mice (arrows), but the scale had to be lowered to 1.0 x10^4 – 1.0 x10^5 p/s/cm^2/sr (blue frame) in order to be able to display these weak signals. **(e)** Two out of three IV-administered BALB/c SCID mice did not show any signals at day 30 in vivo using the standard scale (green frame), however, corresponding **(f)** organ imaging showed small foci of bioluminescence signals in the lungs (arrows).
