# Supplementary material for: Non-invasive imaging reveals conditions that impact distribution and persistence of cells after in vivo administration
Source: Stem Cell Res Ther. 2018 Nov 28;9:332. doi: 10.1186/s13287-018-1076-x (PMC6264053; doi:10.1186/s13287-018-1076-x)
Supplement: Supplementary file 8 — Fluorescence Activated Cell Sorting (FACS) analysis of bone marrow extracts. Green fluorescence analysis of cells harvested from the femurs and tibias of (a) a control mouse that received no cells (b) a mouse that received mMSCs IC display no evidence of ZsGreen+ cells in the bone marrow. (PDF 351 kb) [file 13287_2018_1076_MOESM8_ESM.pdf]

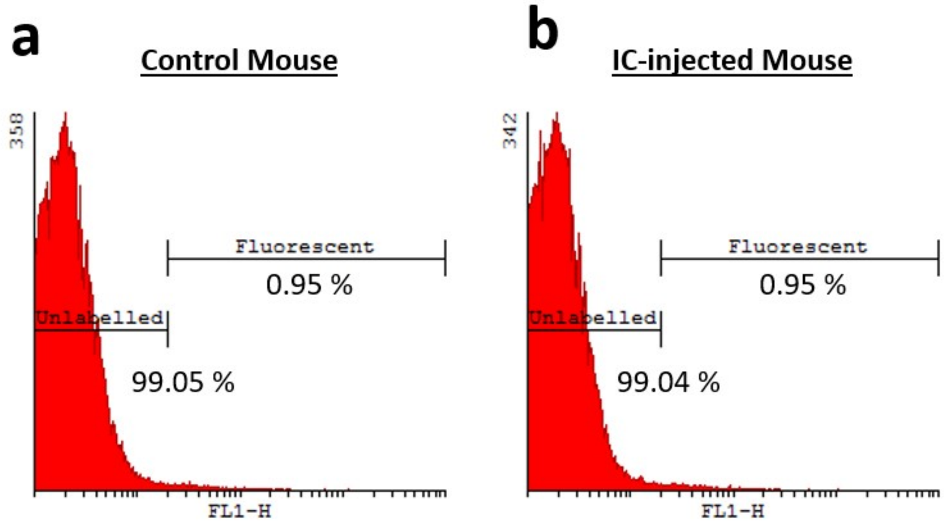

Additional File 8. Fluorescence Activated Cell Sorting (FACS) analysis of bone marrow extracts. Green fluorescence analysis of cells harvested from the femurs and tibias of **(a)** a control mouse that received no cells **(b)** a mouse that received mMSCs IC display no evidence of ZsGreen^{+} cells in the bone marrow.
