# Supplementary material for: Non-invasive imaging reveals conditions that impact distribution and persistence of cells after in vivo administration
Source: Stem Cell Res Ther. 2018 Nov 28;9:332. doi: 10.1186/s13287-018-1076-x (PMC6264053; doi:10.1186/s13287-018-1076-x)
Supplement: Supplementary file 9 — Chromosome analysis of the (a) mMSCs, (b) hBM-MSCs and (c) hUC-MSCs. Whereas mMSCs displayed a grossly abnormal karyotype, the human cells displayed a normal female karyotype. (PDF 422 kb) [file 13287_2018_1076_MOESM9_ESM.pdf]

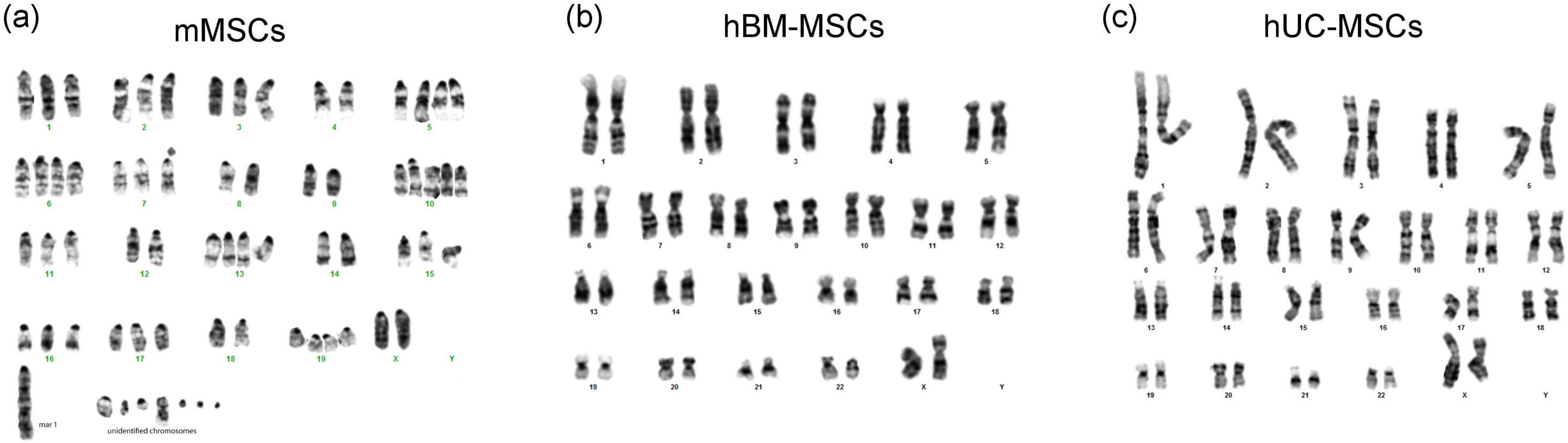

Additional File 9. Chromosome analysis of the (a) mMSCs, (b) hBM-MSCs and (c) hUC-MSCs. Whereas mMSCs displayed a grossly abnormal karyotype, the human cells displayed a normal female karyotype.
