# Supplementary material for: Non-invasive imaging reveals conditions that impact distribution and persistence of cells after in vivo administration
Source: Stem Cell Res Ther. 2018 Nov 28;9:332. doi: 10.1186/s13287-018-1076-x (PMC6264053; doi:10.1186/s13287-018-1076-x)
Supplement: Supplementary file 10 — Ex vivo imaging of organs immediately after administration of 106 hUC-MSC. (a) Intracardiac administration always results in BLI signal originating from organs in addition to the lungs. Intravenous administration, on the other hand, leads to cells lodging predominantly in the lungs. For hUC-MSCs, however, a weak signal was seen in heart, which was particularly noticeable when the lungs were removed from the imaging field. BLI scale: all organs 1.0 × 10^5^–1.0 × 10^7^ p/s/cm2/sr, lungs removed: 1.0 × 10^4^–4.0 × 10^5^ p/s/cm2/sr. (b, c) Relative bioluminescence intensity in each organ as measured ex vivo post (b) IC or (c) IV administration. The signal intensity of mKSCs as shown in Fig. 1d is displayed as a reference. Note that following IV administration, the sum of the signal in organs other than the lungs is generally less than 2% of the total. A break has been inserted in the y-axis to facilitate the visualisation of the data. (PDF 486 kb) [file 13287_2018_1076_MOESM10_ESM.pdf]

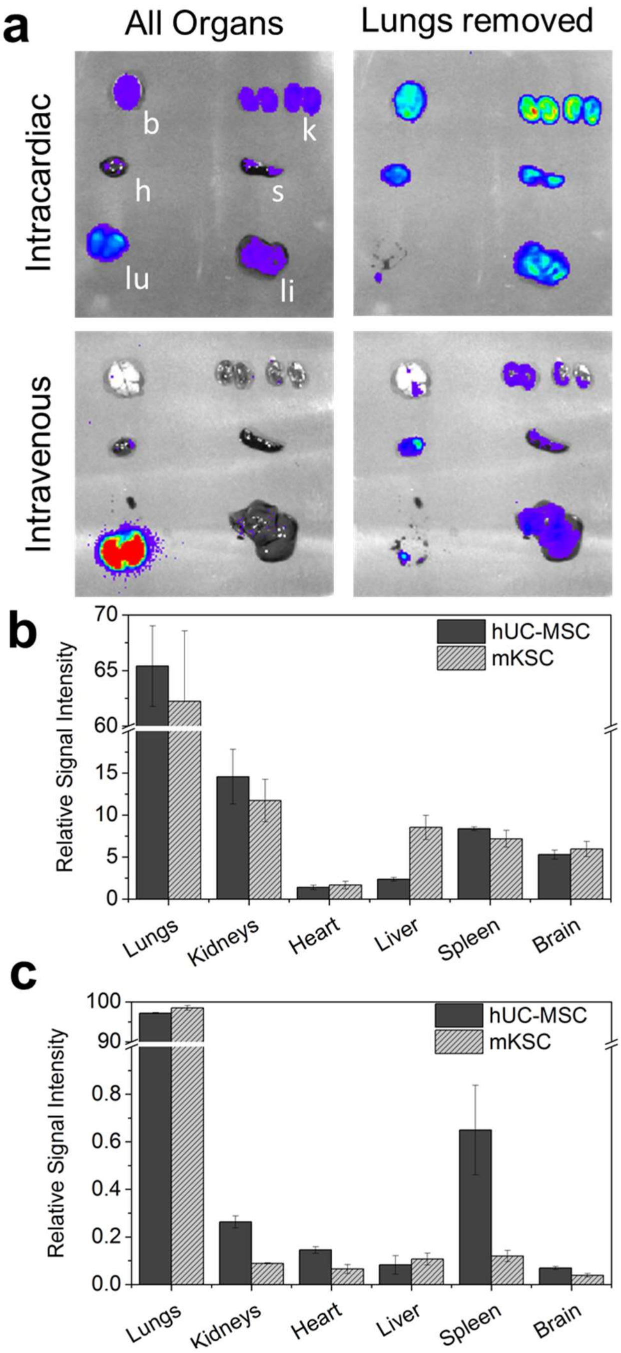

Additional File 10. Ex vivo imaging of organs immediately after administration of 10^6 hUC-MSC. (a) Intracardiac administration always results in BLI signal originating from organs in addition to the lungs. Intravenous administration, on the other hand, leads to cells lodging predominantly in the lungs. For hUC-MSCs, however, a weak signal was seen in heart, which was particularly noticeable when the lungs were removed from the imaging field. **BLI scale:** all organs $1.0x10^5$ – $1.0\ x10^7$ p/s/cm^2/sr, lungs removed: $1.0x10^4$ – $4.0\ x10^5$ p/s/cm^2/sr. **(b-c)** Relative bioluminescence intensity in each organ as measured ex vivo post **(b)** IC or **(c)** IV administration. The signal intensity of mKSCs as shown in Fig. 1d is displayed as a reference. Note that following IV administration, the sum of the signal in organs other than the lungs is generally less than 2% of the total. A break has been inserted in the y-axis to facilitate the visualisation of the data.
